# Studying the Shape Variations of the Back, the Neck, and the Mandibular Angle of Horses Depending on Specific Feeding Postures Using Geometric Morphometrics

**DOI:** 10.3390/ani11030763

**Published:** 2021-03-10

**Authors:** Federica Raspa, Angela Roggero, Claudia Palestrini, Martina Marten Canavesio, Domenico Bergero, Emanuela Valle

**Affiliations:** 1 Department of Veterinary Sciences, University of Turin, 10095 Grugliasco, Italy; federica.raspa@unito.it (F.R.); martina.martencanave@edu.unito.it (M.M.C.); domenico.bergero@unito.it (D.B.); 2 Department of Life Sciences and Systems Biology, via Accademia Albertina 13, 10123 Torino, Italy; angela.roggero@unito.it (A.R.); claudia.palestrini@unito.it (C.P.)

**Keywords:** horse, hay nets, feeding positions, postures, welfare

## Abstract

**Simple Summary:**

Many feeding devices such as hay nets are used to increase the time horses spend feeding on hay. However, when hay nets are used, the horse is often forced to keep unnatural feeding positions. Geometric morphometrics was applied to describe the shape variations of back and neck postures as well as the variations of the mandibular angle according to specific positions adopted during hay feeding: on the ground—control position (CP); neck held 15 ± 3° below withers height with low hay net position (LP); neck held 15 ± 3° above withers height with high hay net position (HP). Our results showed that the back and neck postures as well as the mandibular angle were different in HP compared to CP. Moreover, LP showed that the back posture more closely resembled the shape exhibited by horses feeding from CP; however, no similarity was recorded for neck posture and mandibular angle. Our findings suggest that more attention should be paid when horses keep an unnatural feeding position in comparison to when hay nets are used, since the back and neck postures as well as the mandibular angle can be altered especially when horses are forced to eat with a neck held at 15 ± 3° above the withers.

**Abstract:**

The effects of specific feeding positions upon the horse’s shape variations of the back and neck postures as well as the variations of the mandibular angle have never been objectively studied. For this reason, geometric morphometrics was applied. Six horses, aged 14 ± 8 years (mean ± standard deviation, SD), were video-recorded while using three different feeding positions: on the ground—control position (CP); neck held 15 ± 3° below withers height with low hay net position (LP); neck held 15 ± 3° above withers height with high hay net position (HP). Data were analyzed using principal component analysis (PCA), multivariate analysis of variance (MANOVA), and partial least squares (PLS). A mixed model procedure was applied to evaluate differences in the magnitude of the mandibular angle. Whilst differences between individual horses were confirmed by canonical variate analysis (CVA), PCA analysis showed that a characteristic feeding position could also be identified on a group level. During the HP hay net position, the back and neck postures as well the mandibular angle were different compared to those exhibited by horses feeding from CP. In LP hay net position, the back posture more closely resembled those exhibited while feeding from CP; however, no similarity between LP and CP was found for neck posture and mandibular angle. Since only a few degrees of variation of the feeding position can influence back and neck postures, this aspect should be further investigated. The right compromise between horse welfare, horse safety, and management practices need to be further explored and long-term effects should be investigated.

## 1. Introduction

Under natural living conditions, feral and domestic pasture horses graze and browse for at least 50% of the daytime [1,2], whereas they spend 3–4 h at most on non-foraging-related behaviors [3,4]. According to some horse welfare protocols, fasting should not exceed 4 h [5], and the quantity of forages provided should ensure that the physiological nutritional needs of horses are satisfied [6]. Feeding behavior is linked to the gastrointestinal physiology of the horse since its digestive system is designed to receive small amounts of food in a continuous manner throughout the day [7,8]. Moreover, according to Fraser et al. [9], having the possibility to live a relatively natural life by expressing behaviors close to those performed in natural contexts is key to safeguarding animal welfare. Accordingly, it is important to remember that despite the process of domestication, horses have maintained the species-specific behavioral and physiological needs of their wild ancestors [10]. However, many modern management practices commonly adopted for domestic horse husbandry negatively affect horse welfare. In contrast with natural conditions, horses are often confined to single boxes or small group pens, and provided just two or three daily food rations, thus requiring horses to endure unnaturally long fasting times [11,12]. Indeed, feeding management practices which stray far from natural conditions have been reported to influence horse behavioral repertoires [13] and time budgets [14].

Nowadays, commercial feeding systems, such as hay nets, are used for stabled horses to increase the time they spend feeding on hay with the aim of simulating their natural foraging behaviors [15]. Some studies have evidenced the positive effects resulting from the use of hay nets for horse welfare; for example, Martinson et al. [16] showed how the use of hay nets increases forage consumption times. An increase in time spent exhibiting feeding behavior has also been shown to correlate with a reduction in the expression of stereotypic behaviors—which may indicate the failure of the horse to cope with the stable management practices in use, and which do not at all reflect natural living environments [15,17,18].

However, some negative effects have been highlighted related to the fact that hay nets are often hung high from the ground, thus requiring horses to adopt an unnatural posture for eating [17]. The natural feed intake posture of horses requires that food be on the ground, and it has been reported that feeding on hay from the ground also represents the feeding position most favored by horses [19]. As shown by Ellis et al. [17] and Rochais et al. [20], hay nets are hung high from the ground and hay nets’ positions need to be further investigated since they are responsible for unnatural neck and back postures. Of the various new methodologies developed that aim to avoid subjective evaluations of linear morphometry, geometric morphometrics has been proposed to improve the objectivity and reproducibility of horse postural measures [21]. To the best of our knowledge, no studies to date have applied geometric morphometrics to investigate if specific feeding positions can affect the shape variations of back and neck postures as well as the variations of the mandibular angle. Geometric morphometrics analyzes horse postures through the use of coordinates of homologous anatomical landmarks, and enables minor shape variations in posture to be identified [21,22,23,24]. In particular, geometric morphometrics eliminates size factors, and instead focuses on the shape of individuals, thus permitting the description of overall body morphology [25]. Accordingly, some authors have proposed the use of geometric morphometrics for the characterization of behavioral categories [21,23], and for evaluating the impact of environmental factors or sporting management on health aspects, such as a horse’s back shape or possible vertebral disorders [21,26].

On the basis of this background, the aim of the present study was to apply geometric morphometrics to objectively evaluate the shape variations of back and neck postures as well as the variations of the mandibular angle according to three specific hay-feeding positions (i.e., on the ground—control position (CP); neck held 15 ± 3° below withers height with low hay net position (LP); neck held 15 ± 3° above withers height with high hay net position (HP). We hypothesized that the feeding positions adopted would modify back and neck postures as well as mandibular angle in horses.

## 2. Materials and Methods

### 2.1. Horses and Management

This experiment only involved behavioral observations and noninvasive interactions with the horses. All procedures were performed in full accordance with the current Italian and European regulations (Legislative Decree 26/14—European Directive 2010/63/UE) and with the guidelines for the treatment of animals for behavioral research and teaching [27]. Animal husbandry and care were under the management of private owners. Full authorization was provided by the owners for the researchers to conduct the present study. The horses used in this experiment were not research animals.

Six warmblood mesomorphic horses (denominated: ID 1, ID 2, ID 3, ID 4, ID 5, and ID 6) were involved in the study, of which five were geldings and one was a mare. The horses had a mean age of 14 ± 8 years (± standard deviation, SD) and a body weight of 579 ± 85 kg; their mean height at the withers was 169 ± 8 cm, and they presented a mean body condition score (BCS) of 6 ± 0.5 (according to the nine-point scale developed by Henneke et al. [28]).

All horses were of good general health and regularly checked for dental diseases, hoof care, vaccinations, and parasite control. They were also ridden and schooled regularly. The horses were housed in individual stall boxes (3.5 × 4 m), the floor of which was lined with rubber matting and covered with shavings. The horses were accustomed to eating from hay nets for six months before the start of the study. Their daily feed rations comprised first-cut meadow hay, provided on a 2% Body Weight (BW) basis, divided into two meals (morning and evening), and a cereal-based feed concentrate supplied three times a day (morning, lunch, and evening). Tap water was provided through one automatic drinker. Horses had regular access to paddock areas with some grass on a daily basis.

#### Hay Net Features

The hay nets used in this study were slow-feed HDP (High Density Polyethylene) twine hay nets, with mesh openings of 4 cm and an overall dimension of 1.20 × 1.20 m.

During the video-recording procedures, hay nets were filled with 2 kg of the same first-cut meadow hay that horses were usually fed in their boxes.

### 2.2. Study Setup

#### 2.2.1. Feeding Positions

Hay was presented in three feeding positions, on the ground and at two different heights from the floor. These heights were chosen in order to obtain specific body positions that the animals needed to adopt in order to extract the hay from the nets. However, when a horse is eating from a hay net, it does not maintain a constant feeding position, but its posture frequently changes over time. Indeed, Webster and Ellis [19] showed that stabled horses regularly move between eating from a hay net to eating from the floor. Each horse was evaluated for the three feeding positions during the same day. Thus, the video recordings had to be made for a sufficient length of time to ensure that enough frames could be gathered for assessing the shape variations of back and neck postures adopted by means of geometric morphometrics (as described in Section 2.3).

**Figure 1:**
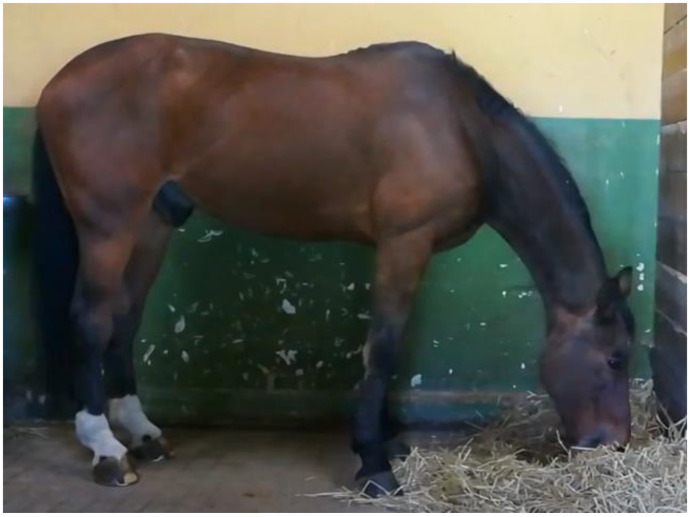
Control position (CP). Hay is on the ground.

- Control position (CP): the hay was offered on the ground, and was considered the natural feeding position (Figure 1).

**Figure 2:**
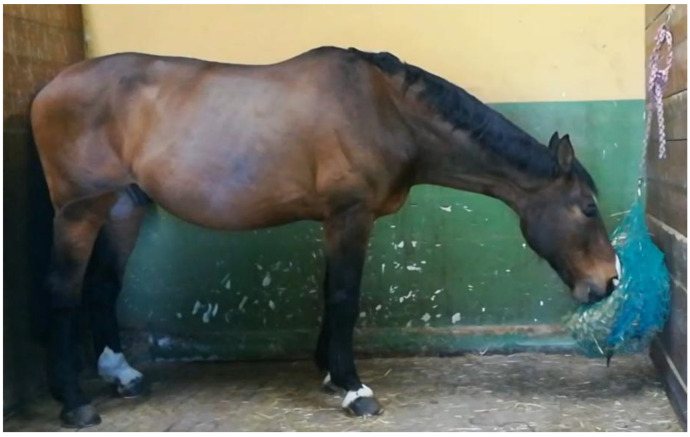
Low hay net position (LP). The bottom edge of the hay net is level with the mid-point of the cannon bone.

**Figure 3:**
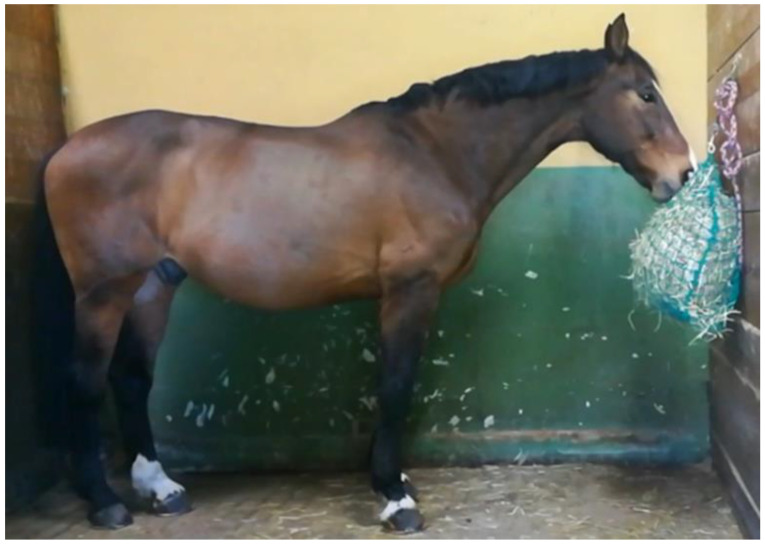
High hay net position (HP). The bottom edge of the hay net is level with the horse’s elbow.

Since the height at withers varied between the participating horses, the other feeding positions set for each horse were established by hanging the hay nets at two different heights determined as follows:

- Low hay net position (LP): the bottom edge of the hay net was level with the mid-point of the cannon bone (Figure 2). In this way, as the horse fed from the net, the neck was slightly below the withers.

- High hay net position (HP): the bottom edge of the hay net was level with the horse’s elbow (Figure 3). In this position, the horse was required to hold his neck slightly above withers height.

Although the horses were already accustomed to eating from hay nets, they were further acclimatized to all the different hay net positions used in this study for one hour/day for five consecutive days according to Ellis et al. [19].

#### 2.2.2. Video Recordings

When performing the video recordings, the horse being assessed was placed in an empty box lined with rubber matting, but without shavings, and equipped with one 2D camera. Video recordings were conducted two hours after the 9 am morning meal using a smartphone (iPhone^®^ 8, Apple, Cupertino, CA, USA) video camera (720p HD of resolution). The device was positioned parallel to the animals, as shown in Figure 1, Figure 2 and Figure 3, at a distance of 2 m and held 1 m above the ground using a specific tripod.

Each horse was fed hay in the three different feeding positions (see Section 2.2.1). Each feeding position was recorded for at least 15 min, and during each video recording, the horse was moved as necessary in order to have the horse stand in the correct position: parallel to the video camera according to the position of the feet relative to a spot placed on the ground.

### 2.3. Data Analysis

All video recordings were analyzed by a single trained operator expert in equine studies using Windows Media Player (version 12, 2009 Microsoft Corporation). The operator selected the best frames of each horse’s feeding positions to subject to the subsequent analysis. Accordingly, at least 20 frames per horse were selected for each feeding position—CP, LP, and HP.

**Figure 4:**
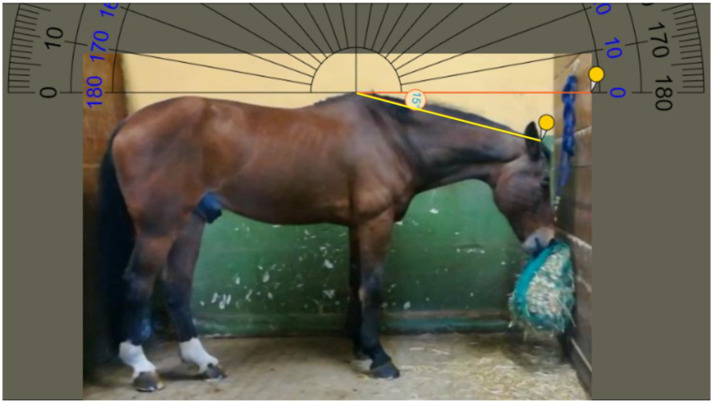
Low hay net position (LP): angle formed between the yellow line running from the withers to the poll of the neck and the reference line crossing the withers and parallel to the ground.

Frames of horses eating in LP were selected as shown in Figure 4: the position of the yellow line (from the withers to the poll of the neck) formed an angle of 15 ± 3° with the reference line (shown in orange) crossing the withers and parallel to the ground.

**Figure 5:**
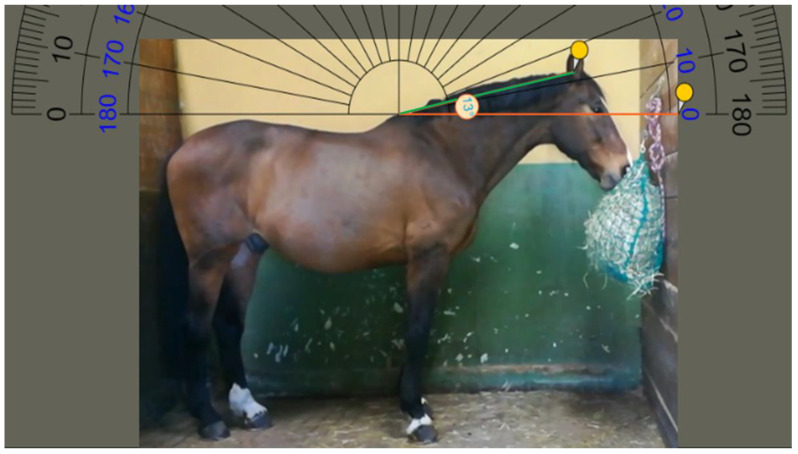
High hay net position (HP): angle formed between the green line running from the withers to the poll of the neck and the reference line crossing the withers and parallel to the ground.

Frames of horses eating in HP were selected as shown in Figure 5: the position of the green line (from the withers to the poll of the neck) formed an angle of 15 ± 3° with the reference line crossing the withers and parallel to the ground.

**Figure 6:**
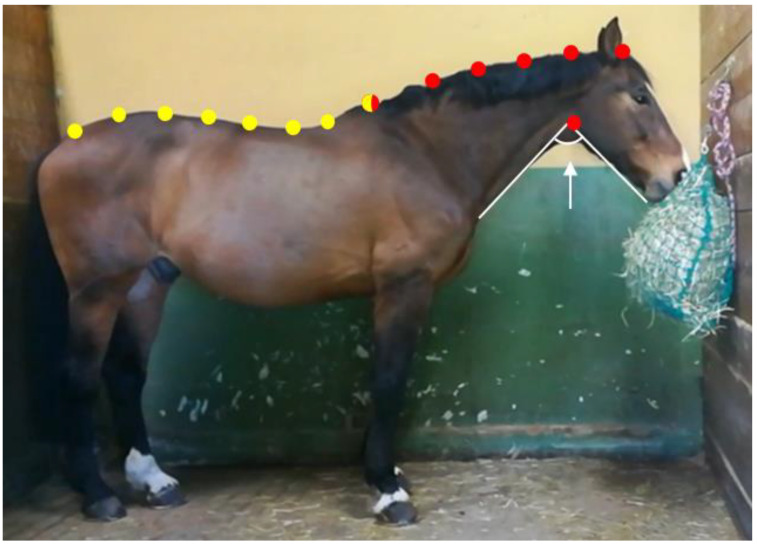
Geometric morphometrics analysis was applied according to the methods described by Tocco et al. [29] and Palestrini et al. [30] to evaluate the shape variations of back and neck postures. Eight configuration points were used for the back (yellow dots) and seven for the neck (red dots). The back and neck were analyzed separately to avoid the Pinocchio effect. The white arrow indicates the angle between the mandible and the underside of the neck that was calculated for tpsRegr v1.41 each feeding position.

Following frame selection, geometric morphometrics analysis was applied to evaluate the overall shape variation of the back and the neck posture of each horse according to each feeding position. For the analysis, the method described by Tocco et al. [29] and Palestrini et al. [30] was applied, in which landmarks and semi-landmarks were coded in tpsUtil 1.76 [31]. Briefly, the point configurations included eight points along the back and seven points along the neck (Figure 6) using tpsDig 2.31 [32]. A different configuration of points was used for the back versus the neck in order to avoid the Pinocchio effect. Regarding the back, points 1, 7, and 8 were coded as landmarks, and points 2 through to 6 as semi-landmarks (see Figure 4). For the neck, points 1, 6, and 7 were coded as landmarks, and points 2 through to 5 as semi-landmarks (see Figure 5). The goodness of fit for each point’s configuration was tested using tpsSmall 1.34 [32].

The data obtained were analyzed by principal component analysis (PCA), multivariate analysis of variance (MANOVA), and partial least square (PLS), as described below. Moreover, the magnitude of the mandibular angle between the head and neck was calculated for each feeding position using tps Dig 2.31 [32], and the data obtained were analyzed as described in Section 2.3.4.

#### 2.3.1. Principal Component Analysis (PCA)

PCA was conducted in tpsRelw 1.69 according to the relative warp analysis methodology proposed by Rohlf, 2017 [32]. Relative warp scores (RWs), RWs 1 and RWs 2, were selected to describe the shape variations of back and neck postures. The group membership accuracy (CP, LG, HG) was evaluated by means of cross-validation. A classification matrix—CVA (canonical variate analysis)—was derived from each shape variation, employing all the RWs which explained 100% of the overall shape variation [29,30].

#### 2.3.2. Multivariate Analysis of Variance (MANOVA)

MANOVA was used to evaluate the amount of shape variation of back and neck postures between the three feeding positions. Back and neck posture landmarks were analyzed separately to avoid the Pinocchio effect. The shape variations of each horse for the different feeding positions were analyzed separately using [33], with 1000 random permutations in the Permutation Tests.

#### 2.3.3. Partial Least Squares (PLS)

Partial least squares (PLS) analysis was performed by means of tpsPLS v1.22 software [31], with 1000 random permutations in the Permutation Tests. PLS permitted the evaluation of shape covariation between back and neck postures and the comparison of the shape variation with the magnitude of the mandibular angle between the head and neck.

#### 2.3.4. Magnitude of the Mandibular Angle

Statistical analyses were performed using JMPpro v15 (SAS Institute Inc., Cary, NC, USA). The feeding position data values were tested to ascertain whether they fitted a normal distribution and normalized by BoxCox transformation. A mixed model procedure was performed, with the feeding position as the fixed effect and the individual horses as the random effect (experimental unit). Least square means were separated using the Tukey’s procedure when a significant *F*-test (*p* < 0.05) was detected.

## 3. Results

The results obtained from the tpsSmall analyses provided the point configurations required to detect the overall shape variation of the back and neck postures according to the feeding positions studied.

### 3.1. Principal Component Analysis (PCA)

**Figure 7:**
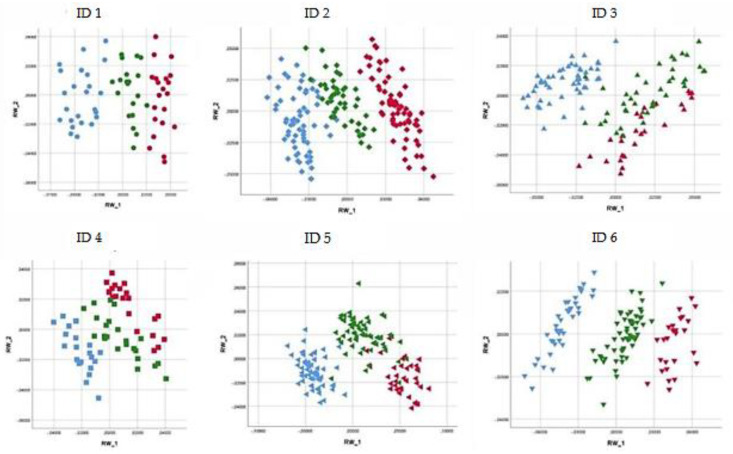
Scatterplot of the first two relative warp scores (RWs) considering the back posture of each individual horse. The three different colors describe the data obtained for three different feeding positions: red, on the ground—control position (CP); green, neck held 15 ± 3° below withers height with low hay net position (LP); and blue, neck held 15 ± 3° above withers height with high hay net position (HP). Principal component analysis (PCA) based on the first two RWs (RW 1 and RW 2) describes the percentage of the overall shape variation: 92.89% for ID 1; 97.02% for ID 2; 95.89% for ID 3; 87.04% for ID 4; 95.71% for ID 5; and 92.78% for ID 6.

PCA showed that the overall shape variations were well defined for each individual horse considering the back and the neck postures according to the three different feeding positions. The percentage of the overall shape variation for the back postures of individual horses was 92.89% for ID 1, 97.02% for ID 2, 95.89% for ID 3, 87.04% for ID 4, 95.71% for ID 5, and 92.78% for ID 6 (Figure 7).

**Figure 8:**
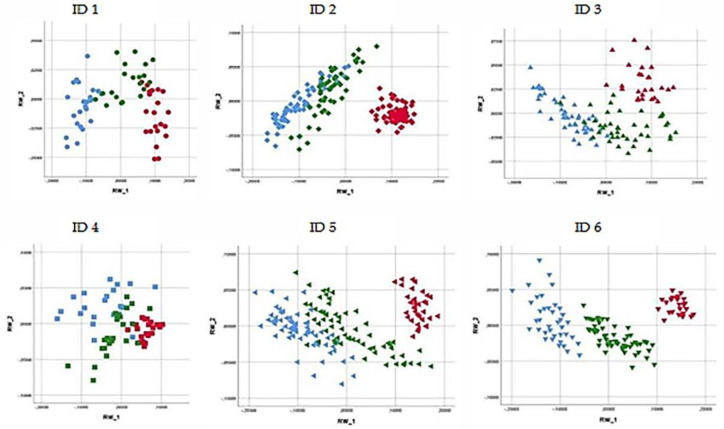
Scatterplot of the first two relative warp scores (RWs) considering the neck posture of the individual horses. The three different colors describe the data obtained for three different feeding positions: red, on the ground—control position (CP); green, neck held 15 ± 3° below withers height with low hay net position (LP); and blue, neck held 15 ± 3° above withers height with high hay net position (HP). Principal component analysis (PCA) based on the first two RWs (RW 1 and RW 2) describes the percentage of the overall shape variation: 96.79% for ID 1; 95.80% for ID 2; 95.25% for ID 3; 90.47% for ID 4; 95.86% for ID 5; and 97.08% for ID 6.

In Figure 8, the scatterplot for the first two RWs describes the neck postures of the individual horses. The percentage of the overall shape variation was 96.79% for ID 1, 95.80% for ID 2, 95.25% for ID 3, 90.47% for ID 4, 95.86% for ID 5, and 97.08% for ID 6.

The results obtained by PCA were confirmed by the CVA classification matrix for the back and the neck postures. Table 1 and Table 2 report the cross-validated results for individual horses (ID 1, ID 2, ID 3, ID 4, ID 5, ID 6) and group membership (Dataset Total) according to the three feeding positions for the back and the neck, respectively. The high percentage of cross-validated results (>80%) for individual horses confirms the PCA outcomes. Moreover, the results obtained show a good level of group membership, indicating the presence of shape variations between individual horses, since when horses were considered together (Dataset Total), the percentage of cross-validated results was lower than when horses were considered individually.

### 3.2. MANOVA

**Figure 9:**
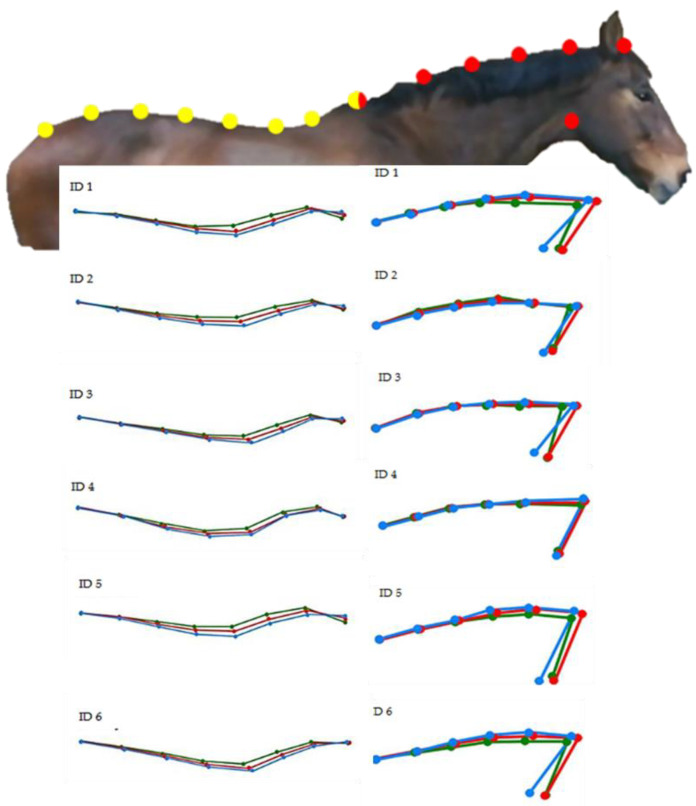
Mean individual shape variations in back and neck postures according to the three different feeding positions: red, on the ground—control position (CP); green, neck held 15 ± 3° below withers height with low hay net position (LP); and blue, neck held 15 ± 3° above withers height with high hay net position (HP).

The results obtained by MANOVA describe the amount of individual shape variation for the back and neck postures according to the three feeding positions (Figure 9). In particular, the mean individual shape variations between the different feeding positions are characterized by the same pattern of variation, demonstrating how geometric morphometrics is able to detect minimal variations in posture.

### 3.3. Partial Least Squares (PLS)

Back and neck data were evaluated separately in order to avoid the change in head position covering the real variation of the shape. Therefore, the relationship between back and neck postures was assessed by means of PLS. PLS analysis was used to evaluate whether the shape covariation between back and neck postures was correlated. The results reported in Table 3 show a high correlation between back and neck postures. In particular, the permutation test value—equal to 0.10%—confirmed the significant correlation between back and neck postures.

### 3.4. Magnitude of the Mandibular Angle

Table 4 shows the results related to mandible angle for each feeding position. The median (25th–75th quantiles) mandibular angle was greatest when horses were fed on the ground. In particular, the mandibular angle in CP—153.25 (145.67–161.43)—was significantly higher than the mandibular angle in LP—113.12 (110.78–121.23). Moreover, the mandibular angle was significantly greater in LP than in HP—97.74 (91.11–102.91).

## 4. Discussion

To the best of our knowledge, an objective investigation into whether feeding horses at different heights from the ground affects the shape of back and neck postures has never been addressed in the literature. To fill this gap, the present study evaluated three feeding positions—CP, LP, and HP—using geometric morphometrics to conduct an objective assessment of the variation of the shape of back and neck postures [34].

Managing horses in a way that reflects natural conditions is key to safeguarding horse welfare [10,35]. As highlighted by Henderson [36], satisfying the physiological and behavioral needs of horses is crucial in order to improve their welfare. It is well known that one of the main factors that negatively affects horse welfare under stabled management conditions is related to feeding practices, such as a diet high in concentrated feedstuffs and low in fiber and long fasting times [4]. For this reason, hay forage diets provided in a hay net are often proposed in order to reduce the risk of long fasting times, with the aim of meeting the natural foraging needs of horses [15,17,20,37,38,39]. Despite the positive hay net use, hanging them high from the ground [17,20] has been recognized as having potential negative effects on back and neck postures, which can, in turn, negatively affect horse welfare. In fact, horses evolved as grazers, eating from the ground, which has also been shown to be the preferred feeding position of horses [19].

Our results showed that the feeding positions studied affected the shape of back and neck postures during the feeding time. PCA showed that the overall shape variations for back and neck postures were well defined for each individual horse according to the three different feeding positions. In fact, as described in Figure 7 and Figure 8, each feeding position was clearly distinguishable because the percentage of the overall shape variation in back and neck posture was always greater than 90%.

Moreover, the CVA classification matrix confirmed the individual differences detected by the PCA analysis (Table 1 and Table 2). Individual variability could be due to several factors, such as individual characteristics (e.g., age and breed) or living conditions [21,40,41,42,43]. Several authors have suggested that environmental, management, and working conditions have a greater impact upon horse postures than aging [44,45]. However, more investigations are needed concerning the impact of different breeds on postures. In particular, Lesimple et al. [40] have shown how stabled horses are continuously subjected to postural modifications induced by the environmental conditions in which they are kept—for example, horses are often fed in buckets fixed on walls or box doors in elevated positions, or they may need to maintain a high head and neck position in order to see out of their box due to high box doors.

Although individual differences were detected between the horses involved in this study, the percentage of cross-validated results showed by the CVA classification matrix (Table 1 and Table 2) was greater than 70%, suggesting that each feeding position could be recognized at the group level.

MANOVA analysis was subsequently applied to describe the amount of shape variation of back and neck postures (Figure 9). MANOVA analysis was carried out considering the back and neck separately in order to avoid the Pinocchio effect. Our findings revealed that although the mean shape of individual horses varied according to each feeding position, back and neck postures showed different patterns of shape variation according to each feeding position. In particular, regarding back postures, the main similarities in shape variation were between CP and LP. This result suggests that a more natural feeding position for the back is achieved—that is, resembling that adopted when feeding from the ground—when the neck is held 15 ± 3° below withers height. This effect on the back depends on the neck posture as described by Lesimple et al. [40]. These authors explained that the extensor muscles of the neck and back in the horse are linked together and responsible for skeletal integrity. Moreover, as a consequence of the neck’s elongation, there is an increased length of the front cantilever of the back [46]. Therefore, lowering the neck generates an extension of the *longissimus dorsi* muscle and, in turn, the entire spine, creating a global “round” posture. This relationship between neck and back postures was also shown in our study by the PLS analysis which revealed that there was a high correlation between back and neck postures, in accordance with studies carried out by other authors [41].

However, it is interesting to note that even if CP and LP positions displayed the main similarities in shape variation in the back, this was not evident in the neck. In fact, our results showed that the horses’ neck postures in LP were more similar to those adopted in HP than in CP. Therefore, even if LP allowed a closer back posture to the natural ground position, this was not found for the neck posture. For this reason, the horse’s neck postures should be studied more deeply according to each feeding position. In fact, as stated by Buchner et al. [47], the mass of the head and neck constitute approximately 10% of the horse’s total body mass, and the large distance of these masses from the body’s center of gravity deeply affects the horse’s whole body as well as its movement. Accordingly, the neck of the horse plays a crucial role in the maintenance of the overall shape variation.

Furthermore, not only did we find that the neck posture was affected by each feeding position, but also that the mandibular angle was significantly wider when horses were fed on the ground: 153.25 (145.67–161.43). Our data showed that when horses were fed in LP—that is, with a neck angle 15 ± 3° below the withers—the magnitude of the mandibular angle was significantly lower compared with that achieved in CP: 113.12 (110.78–121.23). Moreover, when fed in HP—that is, with a neck angle 15 ± 3° above the withers—the mandibular angle was reduced even further: 97.74 (91.11–102.91). Accordingly, both feeding positions affected the magnitude of the mandibular angle, and this is an important result from the welfare point of view. In fact, Zebisch et al. [48] showed that hyperflexion of the neck in ridden horses led to changes in the width of the mandibular angle as a welfare stress factor, since it was associated with an increase in neck circumference and in the thickness of the jaw muscle, with a consequent compression of the larynx, thus reducing air exchange and producing higher flow resistance. Considering the results of the present study, we might hypothesize that feeding that forces horses into a specific position, such as HP, could affect the temporomandibular joint over time. More investigations are needed to clarify the presence of long-term anatomical variations in the temporomandibular joint, which could be risk factors for diseases, such as arthritis.

The results obtained in the present study can be influenced by both the low number of animals and the specific mesomorphic body shape of the warmblood horses. Further studies are needed to evaluate the effects of specific feeding positions in other breeds with different conformational body shape, since different neck length can result in a different front cantilever effect on the back [46].

## 5. Conclusions

Our study showed evidence that different feeding positions are able to modify the shape of back and neck postures, as well as the magnitude of the mandibular angle. When the horse maintained the LP hay net position with the neck 15 ± 3° below the withers, the back posture more closely resembled that exhibited during CP, considered as a natural feeding position. Instead, no similarity was recorded for both the neck posture and the mandibular angle. The overall effect on the shape of back and neck postures and mandibular angle was more evident when the horse was forced with the neck 15 ± 3° above the wither height with high hay net position (HP). Even though the LP hay net position promoted a better back posture, this was not sufficient to maintain a neck posture and a mandibular angle similar to that obtained when a horse was fed from CP. Since hay nets are useful to increase the feeding time consumption, it is necessary to investigate all the postures that a horse may achieve when feeding out of a hay net and identify the height which allows a more natural overall posture.

## Figures and Tables

**Table 1:** The canonical variate analysis (CVA) cross-validated results for back postures of individual horses (ID 1, ID 2, ID 3, ID4, ID 5, ID 6) and group membership (Dataset Total) according to the three feeding positions: on the ground—control position (CP); neck held 15 ± 3° below withers height with low hay net position (LP); neck held 15 ± 3° above withers height with high hay net position (HP). Values are expressed as percentages.

	Results for Back Postures (%)			
Horse	CP	LP	HP	Total
ID 1	100	100	92	97
ID 2	98	100	87	95
ID 3	96	85	100	94
ID 4	100	91	100	97
ID 5	94	95	98	96
ID 6	100	100	100	100
Dataset Total	76	66	92	78

**Table 2:** The canonical variate analysis (CVA) cross-validated results for neck postures of individual horses (ID 1, ID 2, ID 3, ID 4, ID 5, ID 6) and group membership (Dataset Total) according to the three feeding positions: on the ground—control position (CP); neck held 15 ± 3° below withers height with low hay net position (LP); neck held 15 ± 3° above withers height with high hay net position (HP). Values are expressed as percentages.

	Results for Neck Postures (%)			
Horse	CP	LP	HP	Total
ID 1	90	90	100	94
ID 2	100	100	88	96
ID 3	100	85	96	93
ID 4	95	91	86	91
ID 5	100	92	98	96
ID 6	100	100	100	100
Dataset Total	96	83	82	86

**Table 3:** Partial least square (PLS) analysis for each individual horse.

Horse	r *	Cross Set Analysis (%) ^a^	Permutation Tests (%) ^b^
ID 1	0.93	99.60	0.10
ID 2	0.93	99.80	0.10
ID 3	0.86	99.70	0.10
ID 4	0.82	93.70	0.10
ID 5	0.85	99.50	0.10
ID 6	0.92	99.60	0.10

r * correlation index; ^a^ percentage of covariance; ^b^ percentage of correlation.

**Table 4:** Median (25–75° quantiles) mandibular angles for the different feeding positions: on the ground—control position (CP); neck held 15 ± 3° below withers height with low hay net position (LP); neck held 15 ± 3° above withers height with high hay net position (HP).

Feeding Positions	Median (25–75° Quantiles)	SEM ^$^	*p*-Value
CP	153.25 (145.67–161.43) ^A^	0.0003	<0.001 *
LP	113.12 (110.78–121.23) ^B^		
HP	97.74 (91.11–102.91) ^C^		

^$^ Mean standard error of BoxCox transformed values; * statistical significance; ^A–C^ values within a column annotated with different superscript letters are significantly different (*p* ≤ 0.01).

## Data Availability

The data presented in this study are available on request from the corresponding author.
